# Implementing a School-Entry Mandate for the Human Papillomavirus Vaccine: Benefits and Challenges

**DOI:** 10.7759/cureus.62519

**Published:** 2024-06-17

**Authors:** Mohammad S Alzahrani

**Affiliations:** 1 Department of Clinical Pharmacy, College of Pharmacy, Taif University, Taif, SAU

**Keywords:** health policy, cervical cancer, public health, hpv vaccine, hpv infection

## Abstract

Human papillomavirus (HPV), a common sexually transmitted infection, has prompted the development of vaccines to mitigate associated cancer risks, particularly cervical cancer. Regions that have achieved a high rate of vaccination coverage are witnessing a transformative impact on public health, with a notable reduction of up to 30% in the incidence of cervical cancer cases. Emphasizing the broader impact of vaccination on public health, this review investigates the role of a school-entry mandate for the HPV vaccine, aiming to inform decisions about its potential benefits and challenges. With a focus on understanding the significance of vaccination, the review delves into its potential to reduce the physical, emotional, and financial burdens associated with HPV-associated cancers. Implementing school-entry mandates for the HPV vaccine offers benefits such as increased vaccination rates, protection against HPV-related diseases, and long-term health advantages. However, challenges include ethical concerns, parental opposition, and logistical issues. Successful implementation requires clear communication, collaboration, and education, with legal and ethical considerations addressing constitutional rights, liability concerns, autonomy, and equitable access. In summary, the implementation of school-entry mandates for the HPV vaccine offers the potential for increased vaccination rates and the reduction of health disparities. However, this approach is challenged by factors such as opposition, associated costs, and legal and ethical considerations. The decision to mandate the HPV vaccine requires a delicate balance between public health priorities and individual rights, necessitating clear communication, education, and collaborative efforts to address the complexities involved.

## Introduction and background

Human papillomavirus (HPV) is a frequently encountered sexually transmitted infection with the potential to cause various forms of cancer. In the United States, around 34,800 annual cancer cases are linked to HPV, affecting six different forms of cancer [1]. HPV infections account for the vast majority of the estimated 528,000 cervical cancer cases in women globally each year, leading to approximately 266,000 deaths annually [2]. Vaccination stands as one of the most powerful means to avert contagious diseases and safeguard the well-being of the general population. It plays an essential part in decreasing the burden of diseases and preventing their spread within communities. The HPV vaccine is no exception. HPV vaccines have been created to mitigate the risk of HPV infections and associated ailments, specifically cervical cancer. Vaccination at an early age is crucial for optimal protection [1]. Understanding the importance of vaccination against HPV is essential in order to make informed decisions about the vaccine and its potential benefits [2].

It is estimated that around 80% of individuals who are sexually active will acquire HPV at some stage in their lifetime. Although most HPV infections resolve on their own without causing any long-term health risks, some types of HPV can lead to serious complications [3]. There is evidence that cervical cancer, which affects hundreds of thousands of women worldwide each year, is primarily caused by HPV. Furthermore, HPV can cause other types of cancer, including cervical, vulvar, vaginal, anal, penile, and oropharyngeal cancers. These cancers can have a significant impact on individuals and their families, leading to physical, emotional, and financial burdens [1].

The World Health Organization (WHO) has established an ambitious goal to attain a minimum of 90% worldwide immunization coverage against HPV for girls below 15 years by 2030 [4]. Nevertheless, the hesitancy to vaccinate and the low uptake rate present substantial obstacles to the realization of this goal and the eradication of cervical cancer. Most initiatives related to HPV have been formulated to enhance public awareness about HPV infection and its vaccine and to eliminate financial barriers to the utilization of the HPV vaccine [5]. Yet, the school-entry mandate for HPV vaccination remains the most controversial policy. In this review, we will explore the significance of HPV vaccines, provide an overview of the school-entry requirement for HPV vaccination, and discuss the benefits and challenges that accompany such a mandate.

## Review

Role of HPV vaccination

Vaccination against HPV represents a potent strategy for averting diseases linked to HPV and lessening the impact of HPV-associated cancers. The HPV vaccine works by triggering the production of antibodies by the immune system, which can identify and combat the virus. By doing so, the vaccine helps to prevent HPV infection and the development of HPV-related diseases [2]. The vaccination is advised for teens and young adults since it is most effective when given prior to exposure to the virus. Starting at age 11 or 12, the Centers for Disease Control and Prevention (CDC) advises routine HPV vaccination for both boys and girls. Vaccination at this age ensures that individuals are shielded prior to beginning sexual activity and potentially being exposed to HPV [1,3].

The primary benefit of the HPV vaccine is its ability to prevent HPV infections. By targeting the most common high-risk HPV types, the vaccine significantly reduces the risk of acquiring these infections. This, in turn, lowers the chances of developing HPV-related diseases such as cervical, vaginal, vulvar, anal, penile, and oropharyngeal cancers [6]. One of the most prevalent malignancies in women globally is cervical cancer, and HPV is a significant contributor to this condition. By specifically targeting the HPV strains that account for the majority of occurrences, the HPV vaccine has been shown to be quite successful in preventing cervical cancer. Vaccination can significantly reduce the incidence of cervical cancer and related mortality rates [2]. Regions with high vaccination rates have witnessed up to a 30% decrease in cervical intraepithelial neoplasia grade 2+ (CIN2+) [2]. HPV infections can also lead to the development of genital warts. The HPV vaccine offers defense against the HPV strains that cause genital warts in the majority of cases. By reducing the prevalence of genital warts, the vaccine not only enhances the quality of life for individuals but also lessens the strain on healthcare systems [7].

Herd immunity is when a sizable fraction of the population is resistant to a specific infection, which can be brought on by widespread HPV vaccination. This indirectly protects unvaccinated individuals by decreasing the virus's overall spread. Herd immunity is essential for halting the spread of HPV and the diseases it is linked to [2]. The HPV vaccine also offers long-lasting protection against HPV infections. The vaccine provides immunity for at least 10 years, and ongoing research suggests that it may provide protection for even longer [6]. This long-term immunity ensures that individuals are protected during their most vulnerable years.

The HPV vaccine has the potential to have moderate adverse effects, such as soreness, redness, or swelling, where the injection was made [6,8]. Additionally, some people may have a minor fever, a headache, or weariness. These effects are generally short-lived and resolve on their own without any long-term consequences. While rare, some individuals may experience more serious adverse reactions to the HPV vaccine. These can include severe allergic reactions, fainting, or Guillain-Barré syndrome [9]. However, these negative effects are incredibly uncommon, and getting vaccinated has many more advantages than disadvantages.

One of the challenges associated with the HPV vaccine is the presence of misconceptions and vaccine hesitancy. Some people could be reluctant or unwilling to get the vaccine due to worries about its effectiveness and safety [10]. To guarantee universal acceptance and adoption of the vaccination, it is essential to address these worries through education and efficient communication techniques.

The prophylactic vaccines activate the humoral immunity and production of virus-neutralizing antibodies, inhibit viruses from entering into host cells, and induce effective protection against HPV infection. The HPV vaccine guards against the most common high-risk HPV types, although it doesn't cover all strains of HPV. As a result, it is still possible to contract HPV infections or develop disorders linked to the virus that are brought on by other HPV strains that are not included in the vaccination. However, the vaccine's effectiveness in preventing the most prevalent HPV types significantly reduces the overall burden of HPV-related diseases [11]. All HPV vaccines can protect against high-risk HPV types, including HPV16 and HPV18 which cause most HPV cancers [12]. Additionally, a noteworthy aspect of HPV vaccines is their ability to provide cross-protection against other HPV strains. For instance, a study found cross-protection against HPV31 and HPV45 types following the administration of bivalent (HPV16/HPV18) vaccines [13]. Similarly, the quadrivalent HPV vaccine has demonstrated cross-reactive immunogenicity against HPV45 [13]. This cross-protection is a significant factor in the effectiveness of HPV vaccines, as it broadens the range of HPV strains they can protect against, thereby potentially reducing the overall burden of HPV-related diseases. However, the extent of this cross-protection and its clinical implications are areas of ongoing research.

Overview of HPV

HPV is a collection of more than 200 closely related viruses, each of which is identified by a different number. Out of these, the vaginal region is known to be infected by about 40 different strains of HPV. Based on their propensity to cause cancer, these genital HPV strains can be further divided into the following two groups: low-risk and high-risk HPV [8].

Cancer-causing HPV kinds are those that are classified as high risk. The majority of HPV-related malignancies are caused by the two most prevalent high-risk HPV strains, HPV-16 and HPV-18. These cancers primarily affect the cervix but can also occur in other areas such as the vagina, vulva, anus, penis, and oropharynx [14]. Cervical cancer is the most significant cancer associated with high-risk HPV infection. It is estimated that HPV infection is responsible for practically every case of cervical cancer in the globe. Anal, vaginal, vulvar, penile, and oropharyngeal cancers are other cancers that can be brought on by high-risk HPV [15].

Those HPV varieties that do not frequently cause cancer are considered low risk. Instead, they are responsible for the development of benign (non-cancerous) growths, such as genital warts. The two low-risk HPV varieties with the highest prevalence are HPV-6 and HPV-11, which are the main causes of genital warts [8]. Small, flesh-colored lumps called genital warts can develop on or near the anus. While they are not cancerous, they can cause discomfort and emotional distress. Furthermore, low-risk HPV does not protect against high-risk HPV infection, and vice versa [16].

The HPV vaccine is made to guard against both the low-risk forms that result in genital warts and the most prevalent high-risk HPV types that are known to cause cancer. The specific strains covered by the vaccine may vary depending on the brand and formulation. However, the most widely used HPV vaccines target the following HPV types: HPV-16, HPV-18, HPV-6, and HPV-11 [2,7]. In addition to these four types, a few HPV vaccinations also offer defense against other high-risk HPV types, such as HPV-31, HPV-33, HPV-45, HPV-52, and HPV-58 [7]. These additional types are responsible for a smaller proportion of HPV-related cancers.

HPV can be transmitted through various modes. Sexual intercourse, including vaginal, anal, and oral sex, is the main way that the HPV is spread. The HPV virus is highly contagious and quickly spreads from one person to another. Even in the absence of outward indications of an infection or symptoms, HPV can be transmitted from one person to another [1]. An estimated 80% of people who engage in sexual activity will develop HPV at some point in their lives. As a result, it ranks as one of the most prevalent STDs globally [3]. Direct skin-to-skin contact with an area that is infected can potentially spread HPV. This can occur during sexual activity or through non-sexual contact, such as during childbirth or through sharing personal items like towels or razors. During childbirth, an infected mother's infant may contract HPV [17]. This is known as vertical transmission.

Preventing the transmission of HPV is crucial in reducing the prevalence of HPV-related diseases, including cervical cancer [18]. Fortunately, there are several effective strategies for preventing HPV transmission. The most effective way to prevent HPV infection is through vaccination. The HPV vaccine is a safe and effective tool that offers defense against the most prevalent HPV kinds that result in genital warts and certain cancers, such as cervical, anal, and oropharyngeal cancer. Males and females should both get vaccinated, ideally, before they become sexually active [1]. Safe sexual behavior can lower the chance of HPV transmission. This includes frequently and appropriately using condoms during sexual activity. While condoms can provide some protection against HPV, they do not provide complete protection as the virus can infect regions that are not protected by a condom [19]. In addition, limiting the number of sexual partners and engaging in mutually monogamous relationships can reduce the risk of HPV transmission. However, HPV can be transmitted even in monogamous relationships if one partner has been previously infected [20].

Regular screening for HPV-related diseases, such as cervical cancer, is essential for early detection and treatment. Cervical aberrant cell alterations can be found with Pap smears and HPV tests, enabling early detection and cervical cancer prevention [21]. Educating individuals about HPV, its transmission, and its prevention is crucial in promoting responsible sexual behavior and encouraging vaccination. Increasing awareness about the importance of HPV vaccination can help reduce the stigma associated with it and encourage more people to get vaccinated [2]. While HPV can be transmitted through skin-to-skin contact, maintaining good personal hygiene can help reduce the risk of transmission. This includes avoiding sharing personal items, like towels or razors, as well as practicing proper hand hygiene [22].

The prevention of HPV transmission and the burden of HPV-related disorders are both greatly reduced by vaccination. The most prevalent strains of HPV that cause genital warts and some types of cancer can be prevented from infection with the vaccine. The vaccination offers long-lasting HPV protection by immunizing people before they become sexually active [1,7]. Herd immunity is an outcome of vaccination, which also saves those who receive it. When a sizable fraction of the population is resistant to a certain infection, herd immunity arises, which hinders the spread of the virus. The transmission of HPV can be drastically decreased by immunizing a substantial section of the population, which is advantageous to both those who have received the vaccine and those who have not [2]. The HPV vaccine has also been proven to be secure and well-tolerated. Its safety and effectiveness have been shown through extensive research and clinical testing. Common adverse effects, such as soreness at the injection site or a moderate temperature, are typically mild and transient. The advantages of vaccination vastly outweigh the dangers of HPV infection and its possible repercussions [1].

Safety of the HPV Vaccine

The development and evaluation of vaccinations, particularly the HPV vaccine, heavily rely on clinical trials and research investigations. These studies provide valuable information about the safety, efficacy, and long-term effects of the vaccine [23-25]. Ensuring the safety of vaccines is of utmost importance; the HPV vaccine has undergone rigorous safety monitoring through clinical trials and post-marketing surveillance. Adverse events following vaccination are carefully monitored and investigated to assess their relationship to the vaccine [25]. Research studies have consistently shown that the HPV vaccine has a favorable safety profile [24,25]. In a comprehensive surveillance study involving over 180,000 females who have received a minimum of one dose of the HPV vaccine, only two conditions were found to be significantly associated with the vaccine: skin infections (with an odds ratio (OR) of 1.8 and a 95% confidence interval (CI) of 1.3-2.4) and syncope (with an OR of 6.0 and a 95% CI of 3.9-9.2) [24]. The most frequently noted side effects are mild and short-lived, including experiences like discomfort at the injection site, fever, and dizziness [7]. Severe adverse events are infrequent, and the advantages of vaccination significantly surpass any potential risks.

To enhance safety monitoring and various surveillance, there are established procedures to identify and explore any potential safety issues. These systems allow for continuous monitoring of the vaccine's safety profile and prompt action if any new safety signals arise [25]. The majority of individuals receiving the HPV vaccine typically encounter only minor, if any, side effects. These side effects are usually short-lived and naturally subside. The most common side effects include discomfort, inflammation, or redness at the site of injection [24]. They usually last for a few days and can be relieved with over-the-counter pain relievers and applying a cold compress to the injection site. Some individuals may experience a low-grade fever after receiving the HPV vaccine. This is a normal immune response and typically resolves within a day or two. Drinking plenty of fluids and taking over-the-counter fever reducers can help alleviate discomfort. Occasionally, individuals may experience mild headaches or dizziness after receiving the HPV vaccine. These symptoms are usually short-lived and can be managed with rest and over-the-counter pain relievers. In rare cases, individuals may experience mild nausea or vomiting after receiving the HPV vaccine. This is usually temporary and can be managed by staying hydrated and avoiding heavy meals.

While rare, there have been reports of more serious adverse reactions associated with the HPV vaccine. Some of the rare adverse reactions that have been reported include allergic reactions [23]. In extremely uncommon instances, individuals may encounter an allergic response to the HPV vaccine. Symptoms of such an allergic reaction may encompass hives, breathing difficulties, or swelling of the face, lips, tongue, or throat. If any of these symptoms occur after receiving the vaccine, immediate medical attention is recommended. Syncope can occur after any vaccination, including the HPV vaccine. It is more common in adolescents and young adults. To prevent fainting, individuals are advised to sit or lie down for 15 minutes after receiving the vaccine. This allows the body to adjust to the vaccine and reduces the risk of injury from fainting. Guillain-Barré syndrome is an infrequent neurological disorder that has been associated with some vaccines, including the HPV vaccine. However, the risk of developing GBS after receiving the HPV vaccine is extremely low [23]. The advantages of vaccination in preventing HPV-related illnesses significantly surpass any potential risk of GBS.

Recommended HPV Vaccine Schedule

Usually, a number of doses of the HPV vaccine are given over a predetermined amount of time. Depending on the age of the person receiving the vaccine, the recommended schedule could change. The WHO and CDC offer recommendations for the HPV vaccine schedule [26,27]. Starting at age 11 or 12, the CDC advises routine HPV immunization for both males and females. The vaccination can be given to children as young as nine years old. Two doses of the vaccination are advised for those between the ages of nine and 14 years, with the second dose being given 6-12 months following the first. Three doses are advised if the vaccine series is started after the age of 15, with the second dose given one to two months after the first dose and the third dose given six months after the first dose. The WHO advises females between the ages of nine and 14 to have routine HPV vaccinations. Healthcare providers play a pivotal role in guiding individuals through the HPV vaccination process. They offer personalized advice based on an individual's unique health profile and circumstances. Therefore, it is of utmost importance to consult with a healthcare professional when considering the HPV vaccine schedule. This ensures that the vaccination process aligns with the individual's health needs and optimizes the potential benefits of the vaccine.

The HPV vaccine is available in different formulations, and the dosage may vary depending on the specific vaccine being used. The most commonly used HPV vaccines are Gardasil and Cervarix [28]. Gardasil is a quadrivalent vaccine that guards against HPV strains 6, 11, 16, and 18 in addition to two others. This vaccine is approved for both males and females. The recommended dosage for Gardasil is two doses for those between the ages of nine and 14, with the second dose given 6-12 months following the first dose [29]. For those who are 15 years of age and older, there are three doses: the second dose is given one to two months after the first dose, and the third dose is given six months after the first dose. Cervarix, on the other hand, is a dual-protection vaccine that guards against HPV strains 16 and 18. It is only approved for usage in females [30]. The recommended dosage for Cervarix is also two doses for those between the ages of nine and 14, and the second dose should be given 6-12 months following the first dose. For those who are 15 years of age and older, there are three doses: the second dose is given one to two months after the first dose, and the third dose is given six months after the first dose [30].

Overview of school-entry mandates

Despite the promising effects of the HPV vaccine in the fight against cancer, the vaccination rate has been low. The global HPV immunization coverage was estimated at 12.2% in 2018 [31]. The slow uptake of the HPV vaccine has been referred to various factors, including personal as well as external influences [5]. Because the HPV vaccine is recommended at a young age, parental consent is required. Therefore, parental decision-making plays a major role in the HPV vaccine utilization. Nevertheless, the public health impact will largely depend upon vaccine utilization in the general population, especially among groups who are notably affected by HPV-related diseases.

School-entry mandates for the HPV vaccine have become a topic of discussion and debate in recent years. These mandates require students to receive the HPV vaccine before entering school, typically at the middle school or high school level. The goal of these mandates is to increase vaccination rates and protect students from the risks associated with HPV infection [32]. The implementation of school-entry mandates varies from country to country and even within different regions of the same country. In the United States, for example, only four states have laws that require HPV vaccination for school entry [33]. Among these states is Rhode Island, which experienced an 11% increase in HPV vaccine initiation among boys following the implementation of a school-entry requirement [34].

Potential Benefits of School-Entry Mandates

School-entry mandates for the HPV vaccine offer several benefits. First and foremost, they can increase vaccination rates among school-aged children. Data from the CDC in the United States shows that the highest vaccination rate among states is 79% in Rhode Island [35], which is one of the few states that have implemented a school-entry requirement. A dynamic model predicted that with a school mandate, 70% vaccination coverage could be achieved by year 8 compared to 23 years without a mandate [36]. By requiring the vaccine for school entry, more students are likely to receive the vaccine, leading to higher levels of protection against HPV infection [37]. As a result, this can lead to a reduction in the transmission of the virus and a decrease in the prevalence of HPV-related diseases [38].

School-entry mandates can also contribute to the overall public health by creating herd immunity. When a significant portion of the population receives the vaccination, the virus's ability to spread is stopped, and those who cannot get the vaccine for medical reasons are protected. This is particularly important for individuals with compromised immune systems or other medical conditions that prevent them from receiving the vaccine [36,39]. Another potential benefit of school-entry mandates is the potential to reduce health disparities. HPV infection and related diseases disproportionately affect certain populations, including low-income communities and racial and ethnic minorities [40]. Mandates can help ensure that all students, regardless of their socioeconomic status or background, have equal access to the vaccine and the protection it provides.

Mandating the HPV vaccine can have long-term health benefits for individuals and communities. By preventing HPV infections, the risk of developing related diseases later in life is significantly reduced. This not only improves the quality of life for individuals but also contributes to the overall well-being of society [41]. Consequently, the implementation of a school-entry mandate for the HPV vaccine can lead to a reduction in healthcare costs associated with the treatment of HPV-related diseases [32]. By preventing these diseases through vaccination, the burden on healthcare systems is alleviated, and resources can be allocated to other areas of need.

Challenges of School-Entry Mandates

Despite the potential benefits, school-entry mandates for the HPV vaccine face several challenges. One of the main challenges is opposition from various groups, including parents, religious organizations, and individuals who have concerns about vaccine safety and efficacy. This opposition can lead to public backlash, legal battles, and difficulties in implementing the mandates [32]. Challenges arise from public perception and acceptance. Media coverage in Puerto Rico, which adopted a school-entry requirement, was found to be neutral or negative, potentially influencing public hesitancy [42]. Mandating the HPV vaccine may face resistance from parents who have doubts regarding the vaccine's effectiveness and safety [43]. Addressing parental opposition requires effective communication strategies, education about the benefits of the vaccine, and addressing any misconceptions or misinformation that may exist.

Implementing a school-entry mandate for the HPV vaccine requires careful planning and coordination. One of the challenges is the cost associated with implementing and enforcing school-entry mandates. This includes the cost of the vaccine itself, as well as the resources needed to track and verify vaccination records. Schools and healthcare providers need to work together to ensure that the vaccine is readily available, that vaccination records are accurately maintained, and that the mandate is effectively enforced. This may require additional resources and training for school staff and healthcare professionals to ensure compliance with the mandates [44]. Additionally, there may be logistical challenges in reaching all students and ensuring they receive the vaccine. This is especially true for students who are homeschooled or attend alternative education programs [45]. Efforts must be made to ensure that these students have access to the vaccine and are included in the mandate. Mandating the HPV vaccine may also disproportionately affect certain populations, particularly those with limited access to healthcare or who face socioeconomic barriers [46]. It is crucial to address these disparities and ensure that all individuals have equal access to the vaccine, regardless of their socioeconomic status.

Legal Considerations

Implementing school-entry mandates for the HPV vaccine raises important legal and ethical considerations. One of the key legal considerations is the authority of the government to require vaccination as a condition for school entry. In some countries, this authority is clearly defined, while in others, it may be subject to interpretation or legal challenges [32]. It is important to navigate these legal considerations and ensure that the mandate is implemented within the boundaries of the law [47].

One of the primary legal considerations when implementing a school-entry mandate for the HPV vaccine is the protection of constitutional rights. In many countries, individuals have the right to make decisions about their own healthcare, including whether to receive vaccinations. Mandating the HPV vaccine may be seen as infringing upon these rights, leading to potential legal challenges [48]. To address this concern, it is crucial to ensure that any mandate is grounded in a compelling public health interest. This requires demonstrating that the benefits of the vaccine outweigh the potential infringement on individual rights. Additionally, legal frameworks should be in place to allow for exemptions based on medical, religious, or philosophical reasons, where applicable [49].

The legal authority to implement a school-entry mandate for the HPV vaccine varies across jurisdictions. In some countries, the power to mandate vaccines lies with the national government, while in others, it is delegated to state or local authorities. Understanding the legal framework and jurisdictional boundaries is essential when considering the implementation of such a mandate [48]. It is important to ensure that any mandate aligns with existing laws and regulations. This includes considering the process for enacting and enforcing the mandate, as well as any potential penalties or consequences for non-compliance [50].

Another legal consideration is the issue of liability and vaccine injury compensation. Vaccines, including the HPV vaccine, are generally safe and effective. However, there is a small risk of adverse reactions or injuries. In the event that a student experiences an adverse reaction or injury as a result of the mandated HPV vaccine, liability and compensation mechanisms must be in place to address these situations [51]. Jurisdictions that have implemented school-entry mandates for vaccines often have established vaccine injury compensation programs. These programs provide a means for individuals to seek compensation for vaccine-related injuries without resorting to lengthy and costly legal battles [52]. Ensuring the availability of such programs can help address concerns about liability and provide a fair and accessible process for individuals who experience adverse reactions.

Ethical Considerations

Mandating any vaccine raises ethical questions regarding personal autonomy and individual rights. Respecting individual autonomy and informed consent is a fundamental ethical principle in healthcare. Mandating the HPV vaccine for school entry raises questions about the extent to which individuals should have the freedom to make decisions about their own healthcare, including vaccination [53]. Mandates must balance the individual's right to autonomy and informed consent with the goal of protecting public health. Some argue that mandating the HPV vaccine may be perceived as infringing on patient and parental autonomy, leading to political and public hesitancy [41,42]. It is important to address these concerns and ensure that the implementation of a mandate respects individual rights while prioritizing public health [41]. Proponents of mandates argue that they are necessary to protect public health and prevent the spread of HPV-related diseases [54]. They contend that the potential benefits of the vaccine outweigh the infringement on individual autonomy. To address these ethical concerns, it is important to ensure that individuals have access to accurate and comprehensive information about the HPV vaccine. This includes providing education about the risks and advantages of vaccination, as well as addressing any misconceptions or concerns. Informed consent should be obtained whenever possible, and exemptions should be available for those who have valid medical, religious, or philosophical objections [55].

Ethical considerations also include issues of equity and access. Mandating the HPV vaccine for school entry may disproportionately affect marginalized communities who may face barriers to accessing healthcare services. It is essential to consider the potential impact of a mandate on these communities and take steps to ensure equitable access to the vaccine [55]. Efforts should be made to address barriers such as cost, transportation, and language barriers that may prevent individuals from obtaining the vaccine. Collaborating with community organizations, healthcare providers, and schools can help identify and address these barriers [56]. Additionally, providing financial assistance or subsidies for those who cannot afford the vaccine can help promote equity and ensure that the mandate does not exacerbate existing health disparities.

Successful Implementation Strategies

The benefits of mandating the HPV vaccine for school entry outweigh the potential drawbacks. By increasing vaccination rates, protecting against HPV-related diseases, and contributing to herd immunity, a school-entry mandate can have an important bearing on public health. However, it is crucial to address ethical, logistical, and legal considerations while effectively communicating the benefits of the vaccine to parents and the community at large.

To successfully implement school-entry mandates for the HPV vaccine, several strategies can be employed. First and foremost, clear and consistent communication is essential. Parents, students, and healthcare providers need to be informed about the rationale behind the mandate, the benefits of the vaccine, and any potential exemptions or accommodations [35]. Collaboration between schools, healthcare providers, and public health agencies is also crucial. This collaboration can help ensure that students have access to the vaccine, that vaccination records are accurately tracked, and that any challenges or concerns are addressed in a timely manner [54]. Furthermore, education and outreach programs can help address misconceptions and concerns about the vaccine. Delivering precise data on the HPV vaccine's effectiveness and safety can help alleviate fears and increase acceptance among parents and students [57].

## Conclusions

The implementation of school-entry mandates for the HPV vaccine presents a multifaceted landscape of benefits and challenges. While these mandates offer the potential to significantly increase vaccination rates, reduce health disparities, and contribute to herd immunity, they are met with formidable obstacles such as opposition, associated costs, and legal and ethical considerations. The advantages include enhanced protection against HPV-related diseases, the promotion of long-term health benefits, and a potential reduction in healthcare costs. However, addressing ethical concerns, parental opposition, and logistical challenges and ensuring equitable access are crucial for successful implementation. Overall, the decision to mandate the HPV vaccine for school entry requires a balanced approach, considering both public health imperatives and individual rights, with clear communication, education, and collaboration playing pivotal roles in navigating these complexities.
